# Supplementary material for: Direct observation of desorption of a melt of long polymer chains
Source: Nat Commun. 2020 Aug 28;11:4354. doi: 10.1038/s41467-020-18216-y (PMC7455717; doi:10.1038/s41467-020-18216-y)
Supplement: Supplementary file 1 — Supplementary Information [file 41467_2020_18216_MOESM1_ESM.pdf]

Supplementary Information for "Direct observation of desorption of a melt of long polymer chains"

Xavier Monnier[1], Simone Napolitano[2] & Daniele Cangialosi[1,3]

[1]Donostia International Physics Center, Paseo Manuel de Lardizabal 4, 20018 San Sebastián, Spain
[2]Laboratory of Polymer and Soft Matter Dynamics, Experimental Soft Matter and Thermal Physics, Faculté des Sciences, Université Libre de Bruxelles (ULB), CP223, Boulevard du Triomphe, 1050 Brussels, Belgium
[3]Centro de Física de Materiales (CSIC-UPV/EHU) Paseo Manuel de Lardizabal 5, 20018 SanSebastián, Spain

Supplementary Figures

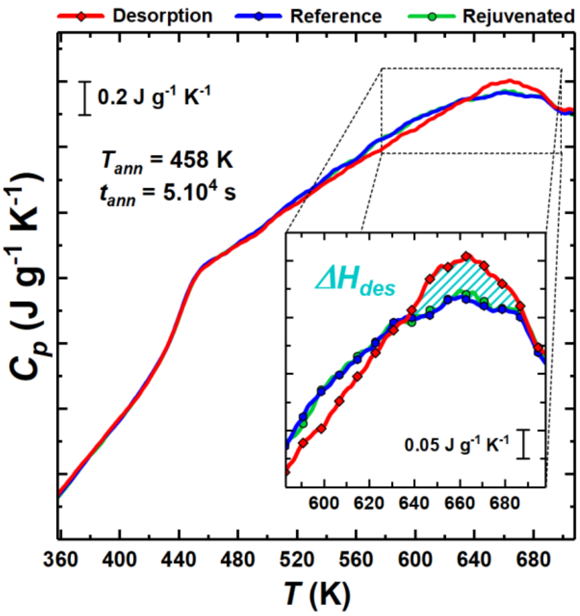

Supplementary Figure 1. Determination of enthalpy of desorption. Specific heat scans on heating at 10^4 K s^{-1} after annealing at the indicated temperature and annealing time (red curve), before adsorption (blue curve) and after desorption (green curve). The inset is an enlargement in the temperature range where desorption takes place. The cyan hatched area shows how the amount of enthalpy of desorption is determined. Specifically, the total enthalpy of desorption measured after a given annealing temperature and time is obtained considering the integral between the desorption curve and that of the reference/rejuvenated curves. The uncertainty on the integration is about 0.5 J/g.

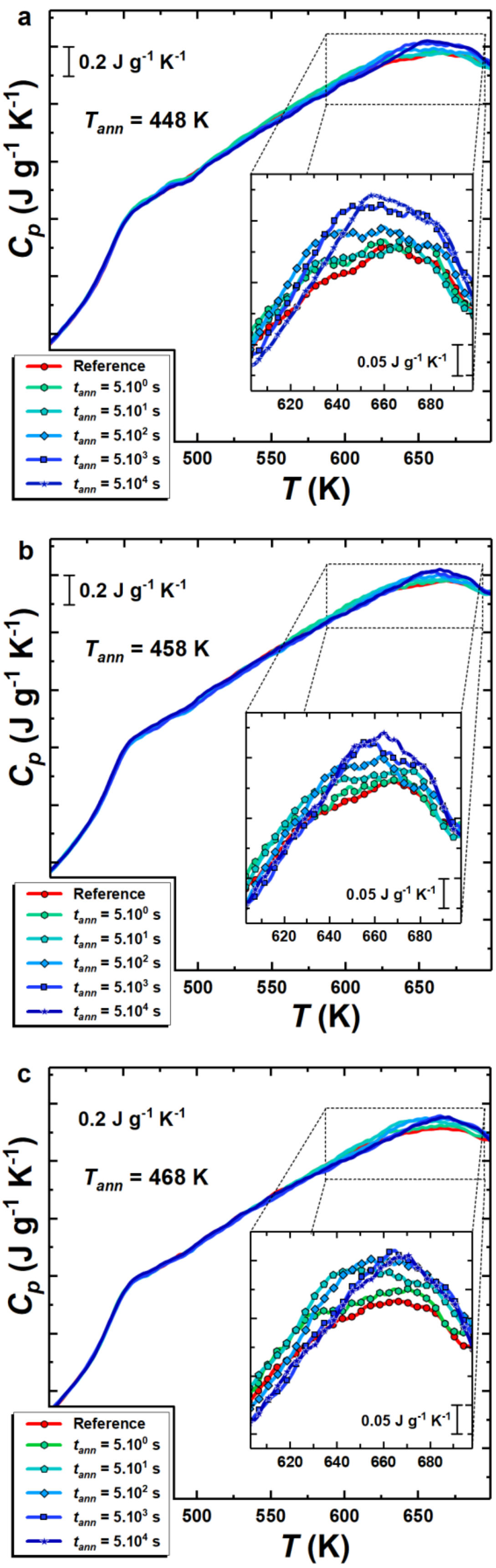

Supplementary Figure 2. Kinetics of desorption. Heating scans at 10^4 K s^{-1} after annealing at (a) 448 K, (b) 458 K and (c) 468 K for various annealing times. The inset is an enlargement in the temperature range where desorption takes place. In all cases, the magnitude of the peak increases with time, until all peaks superimpose at the longest annealing times.

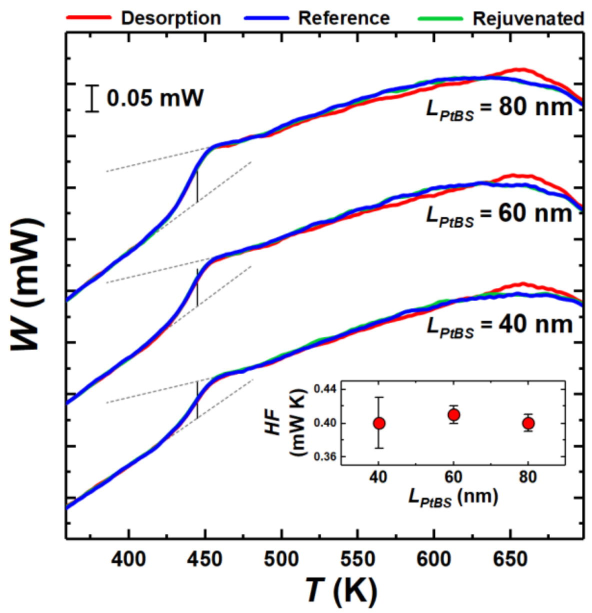

Supplementary Figure 3. Desorption as a function of thickness. Heat flow rate at 10^4 K s^{-1} after annealing at 458 K during 5.10^4 s (red curve), before adsorption (blue curve) and after desorption (green curve) for the three indicated film thicknesses. The dashed grey lines highlight the liquid and glassy lines and the vertical black line depicts the heat flow rate step associated to the 40 nm thick film. The inset shows the heat flow associated to desorption as a function of the film thickness. See also Supplementary Note1

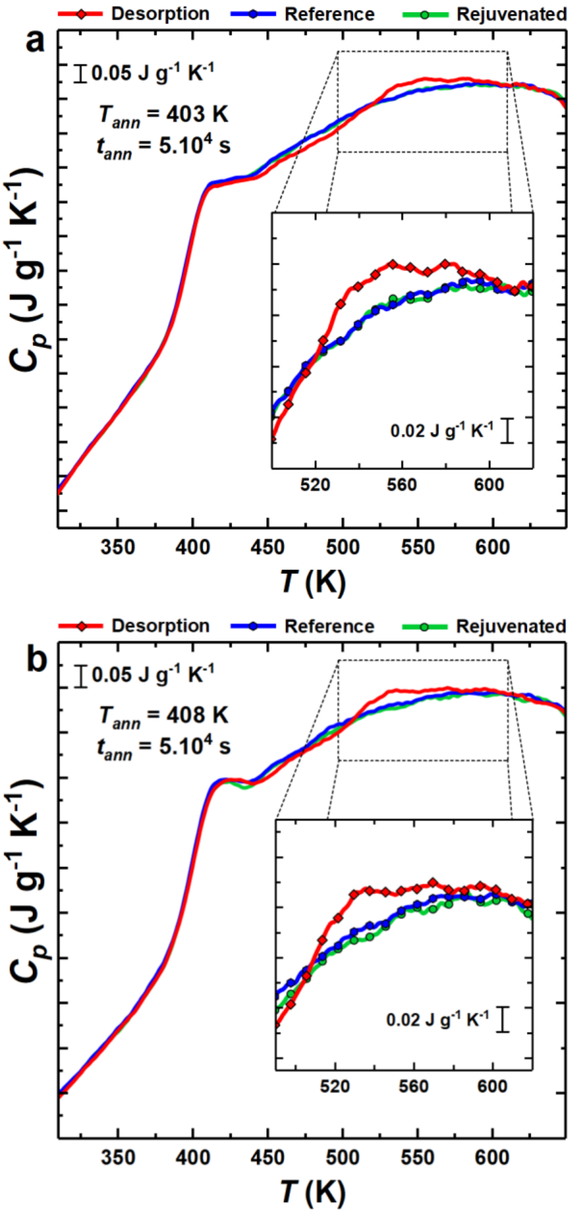

Supplementary Figure 4. Desorption of PS. Heating scans for PS at 10^4 K s^{-1} after annealing at (a) 403 K, (b) 408 K, (c) 413 K and (d) 418 K during 5.10^4 s. The inset is an enlargement in the temperature range where desorption takes place. (e) Enthalpy of desorption of the endothermic overshoot as a function of the annealing temperature. See also Supplementary Note 2.

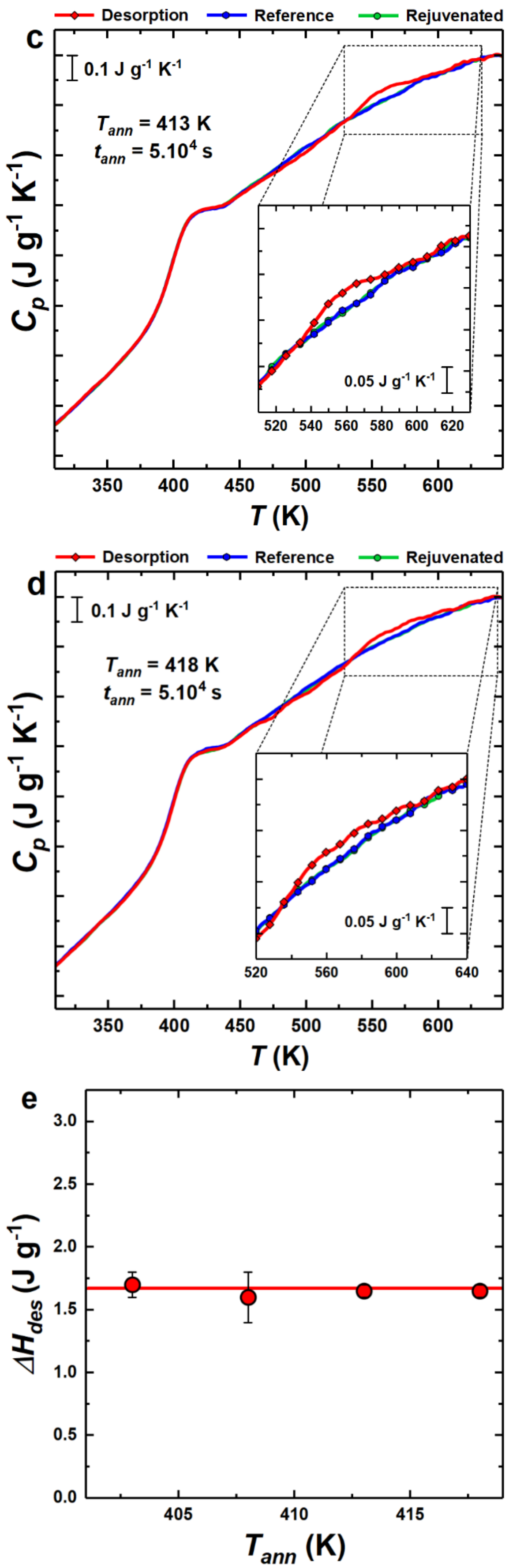
Desorption
Reference
Rejuvenated
c
0.1 J g-1 K-1
T_ann = 413 K
t_ann = 5.10^4 s
0.05 J g-1 K-1
520 540 560 580 600 620
Cp (J g-1 K-1)
350 400 450 500 550 600
T (K)
Desorption
Reference
Rejuvenated
d
0.1 J g-1 K-1
T_ann = 418 K
t_ann = 5.10^4 s
0.05 J g-1 K-1
520 560 600 640
Cp (J g-1 K-1)
350 400 450 500 550 600
T (K)
e
3.0
2.5
2.0
1.5
1.0
0.5
0.0
ΔH_des (J g-1)
405 410 415
T_ann (K)

Supplementary Notes

Supplementary Note1. Supplementary Figure 3 shows the heat flow rate versus temperature of poly(4-*tert*-butylstyrene) (P*t*BS) thin films with various thicknesses ranging from 40 to 80 nm. As P*t*BS was directly spin coated on the backside of the chip, the same sensitive area was covered. This implies that the increase in the heat flow rate step at the glass transition scales with the film thickness. The vertical black line, which defines the heat flow step of a 40 nm thick film, highlights such increase. A different behavior is observed for the endothermic peak related to desorption after identical annealing conditions. In this case, importantly, the amount of heat exchanged during desorption as well as the temperature range of desorption are independent of the film thickness. This is quantitatively highlighted in the inset of Figure 3 that depicts the heat flow related to desorption for each film thickness. This is independent of the film thickness and, therefore, we can conclude that the same amount of polymer is adsorbed regardless of the sample thickness.

Supplementary Note2. To highlight the universal nature of the phenomenon described in the main manuscript with P*t*BS, the adsorption/desorption of polystyrene (PS) has also been investigated. Thin films with thicknesses ranging from 60 to 90 nm were subjected to different annealing temperatures (403 to 418K) during 5.10^4 s. Figure 4 shows the calorimetric response at 10^4 K/s of these films before annealing (reference), after annealing (desorption) and after heating to high temperature the latter samples (rejuvenated). PS thin films undergo a glass transition with a characteristic step in the specific heat capacity at about 400 K. At higher temperatures, an endothermic overshoot is observed in a range essentially independent of the annealing temperature between 520 and 600 K. Similarly to P*t*BS, such overshoot is attributed to the desorption of absorbed polymer chains on the substrate. Importantly, as for P*t*BS, the heat of desorption does not depend on the annealing temperature (see Figure 4e). This result indicates that annealing at different temperatures always delivers the same adsorbed amount.
